# Characterizing the content and quality of internet resources on exercise training in Ehlers-Danlos Syndromes and generalized hypermobility spectrum disorder

**DOI:** 10.1371/journal.pone.0325709

**Published:** 2025-06-26

**Authors:** Jillian Dhawan, Sahar Sohrabipour, Ali Salman Al-Timimi, Brenawen Elangeswaran, Omer Choudhary, Noor Al Kaabi, Megha Ibrahim Masthan, Daniel Santa Mina, Laura McGillis, Wing Ting Truong, Encarna Camacho Perez, Jane Schubart, Mark Lavallee, Timothy Sheehan, Neyha Cherin, Nimish Mittal, Hance Clarke, Rebecca Bascom, Dmitry Rozenberg

**Affiliations:** 1 Toronto General Hospital Research Institute, University Health Network, Toronto, ON, Canada; 2 Division of Respirology, Temerty Faculty of Medicine, University of Toronto, Toronto, ON, Canada; 3 GoodHope Ehlers-Danlos Syndrome Clinic, University Health Network, Toronto, ON, Canada; 4 Faculty of Kinesiology and Physical Education, University of Toronto, Toronto, ON, Canada; 5 Department of Anesthesia and Pain Management, University Health Network, Toronto, ON, Canada; 6 Department of Surgery, Penn State College of Medicine, Hershey, Pennsylvania, United States of America; 7 Department of Public Health Sciences, Penn State College of Medicine, Hershey, Pennsylvania, United States of America; 8 Department of Orthopedics, UPMC Central Pennsylvania, Harrisburg, Pennsylvania, United States of America; 9 Department of Medicine, Penn State College of Medicine, Hershey, Pennsylvania, United States of America; 10 Department of Physical Medicine and Rehabilitation, Penn State College of Medicine, Hershey, Pennsylvania, United States of America; Al Nasiriyah Teaching Hospital, IRAQ

## Abstract

**Background:**

Individuals with Ehlers-Danlos Syndromes (EDS) and Generalized Hypermobility Spectrum Disorder (G-HSD) experience musculoskeletal joint instability, cardiopulmonary manifestations, and functional limitations with online exercise resources commonly utilized. This study characterizes and assesses the content, quality, and readability of websites addressing exercise training for individuals with EDS/G-HSD.

**Methods:**

The first 350 English websites were Googled using search terms “Ehlers-Danlos Syndrome and exercise” and “Ehlers-Danlos Syndrome and physical activity,” targeting educational/instructional sites on exercise training for adults with EDS/G-HSD. Content was assessed using scientific consensus criteria, quality using Modified DISCERN, Global Quality Scale (GQS), and the Patient Education Materials Assessment Tool (PEMAT), and readability using Flesch-Kincaid Grade Level (FKGL) and Flesh-Reading Ease Scores (FRES).

**Results:**

78/350 unique websites were included, most from industry organizations (37%) and personal commentary (24%). The mean content score was moderate 13.8 ± 4.4/25. The content most discussed included: short/long-term benefits of muscle strength, resistance training, and generalized exercise safety considerations. Median modified DISCERN and GQS scores were 4/5 IQR [3–4] and 3/5[2.3–4], respectively. Mean PEMAT understandability and actionability scores were 85% ± 12% and 69% ± 23%, respectively. Average FKGL was 11.0 ± 2.7 and FRES was 43.6 ± 7.2. Moderate-strong Spearman correlations were observed between total content scores and GQS (rho = 0.76) and DISCERN (rho = 0.52), p < 0.001 for both.

**Conclusion:**

Website content varied, most addressing general safety recommendations and multiple training modalities. While quality was moderate-to-good, future resources should focus on simplified language, actionable guidance, and visual aids. Incorporating practical examples of daily activities, injury prevention strategies, broader benefits like cardiovascular health, and psychological support can empower safe and confident exercise training.

## Introduction

Ehlers-Danlos Syndromes (EDS) encompasses a group of genetic connective tissue disorders marked by variable degrees of skin hyperextensibility, joint hypermobility, and tissue fragility [1]. EDS is estimated to affect 1/5,000–1/20,000 people worldwide, commonly involving the musculoskeletal, gastrointestinal, and cardiorespiratory systems [2]. The 2017 EDS classification system recognizes 13 EDS subtypes; whereas generalized hypermobility spectrum disorder (G-HSD) is used to describe patients with EDS-like musculoskeletal sequelae, but who do not meet the additional diagnostic criteria for EDS [3].

As EDS/G-HSD are chronic conditions, exercise and rehabilitation are important for disease management, physical and psychological well-being [4–12]. Exercise training involves regular, structured aerobic, resistance, stability, and balance exercises with the intended purpose of improving physical fitness, daily function and quality of life [4]. Physical rehabilitation emphasizes development of muscular strength [13], proprioception [14–16], and postural exercises for lumbar spinal stabilization and trunk muscle endurance [17]. Given that pain, fatigue, and fear of injury are common barriers to exercise in individuals with EDS/G-HSD, populations-specific information for patients is important to ensure safe exercise and to manage kinesiophobia, which is defined as fear of movement [14]. For preservation of muscle strength, endurance, and aerobic capacity, long-term exercise training is recommended. Rehabilitation programs have increasingly incorporated virtual delivery methods improving accessibility and allowing individuals to continue their exercise routines in their homes [18].

The Internet has been increasingly used for easier access to health information. The International Telecommunication Union estimates that between 56% to 79% of United States (U.S.) users currently obtain health information online for health management [19]. In shifting towards a more patient-centric delivery care model, the ability of individuals to access and understand online health information with minimal costs can promote greater involvement in medical decision-making and improve quality of life [20]. However, online health-related information may be erroneous, outdated, or incomplete, which may pose safety risks [21]. Given the hybrid nature of many exercise training programs for individuals with EDS [22], virtual care promotes continuity of care. Thus, it is important for individuals with EDS/G-HSD to have trust in the content and quality of online resources that may help supplement their in-person and virtual care visits. We believe this paper can serve as a useful reference for both individuals with EDS/G-HSD and health care providers, who may wish to direct their patients to reliable, evidence-informed online resources.

Two small studies evaluated internet resources and exercise/physical activity for EDS patients [22,23]. The first study interviewed 30 persons with EDS sought to understand the use of online support groups to mitigate pain [23]. The second study interviewed 6 young adults with joint hypermobility and chronic pain to explore their internet use and experiences [24]. The focus on pain management did not extend to include disease management or rehabilitation strategies such as exercise training, energy conservation strategies, musculoskeletal concerns, or nutritional support.

Given the high prevalence of internet use and gaps in the literature, our objectives of the present study are to characterize and assess the content, quality, and readability of websites related to exercise training for the EDS/G-HSD community. This work is timely and relevant given the evolving evidence that exercise interventions may help improve daily function and quality of life in this population [4,22,25]. Due to the limited in-person care options and the hybrid nature of many EDS programs, our work may help health care providers identify trustworthy online resources to recommend to patients and support patient-centered care in both virtual and in-person settings.

## Methods

### Study overview

An internet search using the U.S. Google search engine was conducted using two separate search terms “Ehlers-Danlos Syndrome and exercise” and “Ehlers-Danlos Syndrome and physical activity”. Google was used since it is currently the largest search engine. The U.S. version allows our work to reach a greater EDS/G-HSD population with a more extensive network of clinics and specialists. This also aligns with the U.S.-based International Consortium on Ehlers-Danlos Syndromes and Hypermobility Spectrum Disorders [26]. This study did not require research ethics board approval as it exclusively analyzed publicly available internet resources using standardized evaluation tools. No human participants, identifiable personal data, or intervention-based research were involved.

### Search strategy and study selection

**Fig 1:**
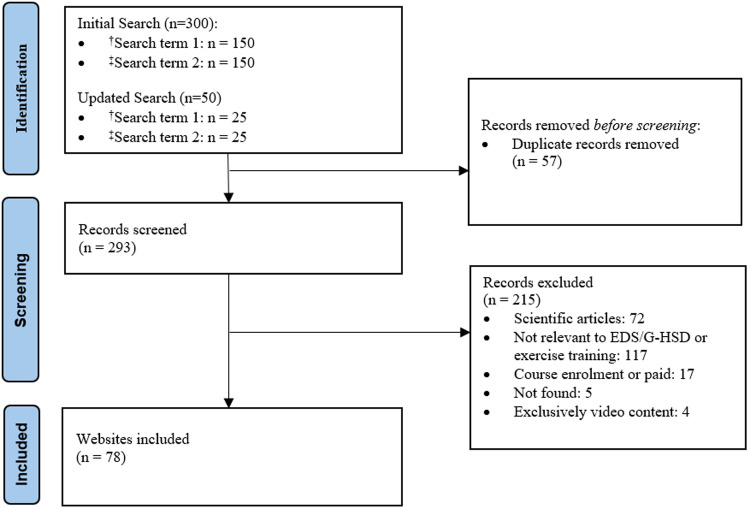
Flow diagram of websites included*. ^†^Search term 1: “Ehlers-Danlos Syndrome and exercise”. ^‡^Search term 2: “Ehlers-Danlos Syndrome and physical activity”. *On July 14, 2023, the first 150 identified English websites for each of the two search terms in Google were selected for the study (n = 300). An updated search was conducted on July 2, 2024, to identify any new or relevant websites, assessing the first 25 websites for each search term.

On July 14, 2023, the first 150 identified English websites for each of the two search terms in Google were selected for the study (n = 300) (Fig 1). We included the text from videos embedded within websites as part of content, as videos are a common educational/informational tool and often assist audiences to retain information and encourage participation. An updated search for the first 25 websites of each search term was conducted on July 2, 2024, to identify any new or relevant websites. While there are no other studies that assess the content, quality, and readability of exercise training among individuals with EDS/G-HSD, there are other studies assessing online resources for other health conditions, including several studies from our group [27–30].

A U.S Internet Protocol (IP) address was used to conduct searches using the Uniform Resource Locator (URL) for Google (www.google.com). Browser history and cookies were cleared. The first reviewer (J.D) assessed website eligibility, which was verified by secondary reviewers (S.S, A.S.T, B.E) using the following criteria:

The inclusion criteria were: (1) educational and/or instructional websites on exercise training for adults (≥ 18 years old) with EDS or G-HSD; (2) websites must describe the concept of exercise training, physical activity and/or provide examples of exercises intended to improve health outcomes in individuals with EDS/G-HSD. The exclusion criteria were: (1) non-English language content; (2) duplicate websites; (3) exclusively video-based; (4) websites that do not target the EDS and/or G-HSD population; (5) websites that lack an educational and/or instructional component; (6) scientific articles; and (7) websites that require a fee or healthcare provider credential to access.

### Characterization of websites

The following information was obtained from each eligible website (and hyperlinked video): (1) date of publication (including date of most recent update); (2) country of origin; (3) website category (defined below); (4) website type (educational or instructional); (5) target population (all 13 EDS subtypes and G-HSD).

Instructional websites were defined as any website that included an individual-guided exercise component addressing at least one of the following exercise training modalities: endurance/aerobic training, resistance/strength training, stretching/flexibility, balance/proprioception exercises with specific instructions or examples. Educational websites were defined as any websites that defined the key components of exercise training, including education, behavior change, evaluations and/or outcomes, as previously described [31].

Websites were classified into five main categories, similar to Da Silva et al [27]: (1) scientific resources (i.e., academic institutions or government organizations); (2) foundation/advocacy organizations; (3) news or media articles; (4) industry or for-profit organization; or (5) personal commentary (i.e., blog post).

### Website evaluation

#### Website content.

The content was developed based on recent scientific consensus recommendations obtained from clinical trials and review articles [13,15–17,32–40].

Content was scored 1 point for “yes” (indicating this item was adequately addressed) or 0 points for “no” (indicating this item was not appropriately addressed).

#### Website quality.

**Modified DISCERN Score** (S3 Table in S1 File) [41] A standardized quality index of consumer health information that allows healthcare professionals, patients, and the general population to evaluate the quality of health information. The modified score is a 5-point Likert scale with higher scores indicating higher reliability (greatest quality).

**Journal of the American Medical Association (JAMA) Benchmarks** (S4 Table in S1 File) [42] uses four criteria to qualitatively assess websites (authorship, attribution, currency, and disclosure).

**Global Quality Scale (GQS)** (S5 Table in S1 File) [43] uses a 5-point Likert scale to assess website accessibility, quality, overall flow of information, and usefulness of a website to patients with higher scores representing excellent quality, and deemed very useful for patients.

**Patient Education Materials and Assessment Tool for printable materials** (PEMAT-P) (S6 Table in S1 File) [44]: Consists of 17 items to measure understandability and 7 items to measure actionability.

#### Website readability.

**Flesch-Reading Ease Score (FEAS)** [45]: It measures the readability of the text on a scale from 0 to 100 based on the average sentence length and number of syllables per word. A higher score indicates easier readability (0–30, very difficult; 30–50, difficult; 50–60, fairly difficult; 60–70, standard; 70–80, fairly easy; 80–90, easy; 90–100, very easy).

**Flesch-Kincaid Grade Level (FKGL)** [46]: It provides an estimate of the U.S. grade level of education needed to understand a particular text. It is based on the average sentence length and the average number of syllables per word.

The review of website content, quality, and readability level was conducted by two independent reviewers and agreement assessed between primary reviewer (J.D) and secondary reviewers (AS.A, B.E, and S.S). Discrepancies between DISCERN, JAMA Benchmarks, GQS, and PEMAT scores was addressed by assessing each reviewer’s rationale, re-examining the instrument’s guide to clarify how each item was intended to be rated, and assigning a consensus score between the two reviewers for each website. If consensus was not reached, a third reviewer (DR) with expertise in exercise and physical activity in EDS helped resolve any discrepancies.

### Statistical analysis

R Studio version 4.1.0 was used for statistical analysis. Descriptive statistics are reported as proportions (n, %) and mean ± standard deviation or median [IQR] based on data distribution. Spearman correlations examined the relationship between website content and quality/reliability metrics. Statistical significance was set at p < 0.05 for all analyses. Microsoft Excel was used to create the figures.

## Results

### Website characteristics

78 unique websites met eligibility criteria (Fig 1) and their rank in the Google search engine are shown in S1 and S2 Tables in S1 File. Website characteristics are summarized in Table 1, with the two most common categories being industry organizations (n = 29, 37%) and personal commentary (n = 19, 24%). Forty-nine (59%) of websites intended for an EDS population, 12 (15%) for G-HSD, and 20 (26%) for both groups.

**Table 1:** Website characteristics.

Website Category	
Industry/for profit websites	29 (37%)
Personal commentary (blog)	19 (24%)
Foundation/advocacy organizations	15 (19%)
Scientific websites	15 (19%)
**Website Type**	
Educational	64 (82%)
Instructional	14 (18%)
**Target Population **(some websites had both)	
EDS	46 (59%)
Both	20 (26%)
G-HSD	12 (15%)
**Continent of Origin**	
North America	50 (64%)
Europe	18 (23%)
Australia	6 (8%)
Unknown	4 (5%)
**Content Total Score (/25)** ^a^	13.8 ± 4.4
**Modified DISCERN Total Score (/5)** ^b^	4 [3–4]
**Global Quality Score (/5)** ^b^	3 [2.3-4]
**JAMA Benchmark Total Score (/4)** ^c^	3 [2–3]
**PEMAT – Understandability (/17)**	85% ± 12%
**PEMAT – Actionability (/6)**	69% ± 23%
**Flesch-Kincaid Grade Level**	11.0 ± 2.7
**Flesch-Reading Ease Score**	43.6 ± 10.0

Data are shown as n (%), median [interquartile range], or mean ± SD. The content total score was based on a predefined scoring system of 25 key components of EDS/G-HSD and exercise training.

**Abbreviations**: EDS: Ehlers-Danlos Syndrome; G-HSD: Generalized Hypermobility Spectrum Disorder; GQS: Global Quality Scale;

^a^ Content total score rated on a scale from 0–25 with higher scores indicating greater content scores.

^b^ Modified DISCERN and GQS total score rated on a scale from 1–5 with higher scores indicating greater quality.

^c^ JAMA total score reported on a scale from 0–4 with higher scores indicating greater quality.

^d^ Flesch-Kincaid Grade Level with higher scores indicating greater reading difficulty.

Note: The percentage values have been rounded, thus, may not add up to 100%

### Website content

Exercise training and physical activity content varied significantly across websites with a mean score of 13.8 ± 4.4 out of a maximum of 25 items listed, as shown in (Table 2) [13,15–17,32–40]. The most common modalities covered were resistance training (88%), exercise safety recommendations (83%), equipment (79%), muscle strength (78%), and exercise intensity (64%). For example, resistance training emphasized using free weights, body weights, and core stability to enhance muscle strength. Common safety recommendations were to consult a healthcare provider, exercise under supervision, progress gradually, adapt for any injuries, seek medical attention for medical issues, and prioritize rest. Notably, 63% of the websites highlighted exercises to avoid, including high-impact activities, movements that increase pressure on locked joints, and contact sports for those with vascular EDS. Equipment needs included the use of exercise tools (weights, treadmill), mobile technology to monitor activity, and aids like footwear, braces, and neuromuscular taping to reduce pain and enhance safety.

**Table 2:** Criteria for content scoring of websites on exercise training.

Category	Criteria	Examples	Websites (n = 78)
**Definition (/1)**	Exercise Training or Physical Activity [32]	Structured activity that is aimed at improving physical fitness or health, or any activity that requires skeletal muscle movement and/or increased energy expenditure	64 (82%)
**Short and long-term benefits (/7)**	Physical Function [13,15,16,33,34]	Physical health, exercise capacity, tolerance, fitness, endurance, stamina, ability to exercise, functional exercise capacity (i.e., 6MWT)	42 (54%)
	Pain management [13,15–17,33–36]	Pain intensity (i.e., using pain measurement tools)	54 (69%)
	Quality of Life [13,15–17,33–36]	Daily function, mental health (i.e., using questionnaires) (depression, anxiety)	44 (56%)
	Muscle Strength [33,36]	Muscle conditioning/deconditioning, muscle atrophy	61 (78%)
	Joint Stabilization [36]	Joint strength, stability, integrity	51 (65%)
	Cardiovascular fitness [7,17]	Decreased risk of hypertension, hyperlipidemia, diabetes, overweight/obesity, cardiovascular disease^8^	14 (18%)
	Fatigue [7,8,33]	Tiredness, energy levels^8^	18 (23%)
**Exercise training modalities (/4)**	Endurance/Aerobic Training [22,33,35]	Cardiovascular activity, walking (ground-based or on a treadmill), cycling (stationary or outdoor), swimming, running, rowing, Nordic walking, etc. (+/- interval training), marching, toe and heel tapping, reaching arms and legs to side	53 (68%)
	Resistance/Strength Training [13,33,35,37,38]	Training with free weights, resistance bands, resistance machine or body weight, etc. (+/- interval training), core stability	69 (88%)
	Stretching/Flexibility Exercises [22,35,37]	Dynamic movements/range of motion exercises to reduce pain and as warmup to aerobic and strengthening exercises (Thoracic mobility, upper and lower extremities, Tai Chi, mindfulness meditation, Yoga)	42 (54%)
	Balance/Proprioception Exercises [15–17,22,33,37]	Body alignment, awareness, and posture (neuromuscular stabilization, spine stabilization)	49 (63%)
**FIT (/3)** ^*^	Frequency	Exercise progression based on FIT principle	22 (28%)
	Intensity		50 (64%)
	Time		20 (26%)
**Safety Considerations (/6)**	Exercise Safety Recommendations [33]	Consult with healthcare provider, supervision by caregiver/partner in home environment, exercise safety screening tool, gradual progression, exercise adaptions for those with injuries, seek medical attention for exercise-induced injuries (i.e., dislocations, severe pain, new/worsening joint instability, shortness of breath, neurological symptoms), adequate rest and recovery	65 (83%)
	Monitoring Exertional Tolerance [6]	Heart rate, Perceived Exertion Scale (i.e., BORG scale)	13 (17%)
	Medication use [39]	Effect of medications for main EDS symptoms and comorbidities (i.e., nonsteroidal anti-inflammatory drugs, muscle relaxants, antidepressants, cardiovascular and pulmonary medications)	22 (28%)
	Falls Risk	Loss of balance	48 (62%)
	Exercises to Avoid	Pressure on locked joints (weightlifting), high impact (repetitive jumping, running) exercises, contact sports for vascular EDS (i.e., football)	49 (63%)
	Other [5,6,22]	Cardiovascular (hypotension, dizziness, cardiac issues), musculoskeletal (arthritis, low back pain, acute injury, overuse injury), diabetic complications (hypoglycemia), comorbidities (postural orthostatic tachycardia syndrome, craniocervical junction instability, dysautonomia, pulmonary manifestations)	37 (47%)
**Educational aspects of exercise training (/4)**	Exercise self-efficacy [22,34–36]	Goal setting, adherence/compliance, self-management strategies, exercise diary	31 (40%)
	Equipment [5,11]	To exercise (i.e., treadmill, stationary bike, bands, weights), to monitor activity (smart watch, mobile app), to ensure safety/reduce pain/aid (footwear, braces/splints/orthotics to prevent joint dislocation, grips, seating appliances, neuromuscular taping)	62 (79%)
	Sources of Motivation [23,40]	Family/friends, technology, EDS support groups/online communities	22 (28%)
	Other [33]	Hydration, sun protection, weather, air quality health index, nutrition/diet, overcoming kinesiophobia, (i.e., through cognitive behavioural therapy)	30 (38%)
**Overall (/25)**			

Note: Relevant components of the table were adapted from Da Silva et al [27] (original sources: 2013 PR guidelines and the 2021 PR international consensus document [47,48]). Individual clinical trials exploring exercise, rehabilitation, and physiotherapy in people with EDS are referenced in Table 2 criteria and are summarized within review articles by Buryk-Iggers [4], Reychler [10], Palmer [8], Smith [5] and colleagues.

* FITT principle: Type is captured under the content item ‘Exercise Training’ as additional examples.

Content elements on websites that were less commonly addressed were stretching/flexibility exercises (54%), motivational strategies (28%), exercise duration (26%), cardiovascular fitness (18%), and assessment of exertional tolerance (17%). Exertional tolerance assessments included modalities such as the ‘talk test’ to gauge if the intensity allows for simple conversation or employing the BORG Rating of Perceived Exertion scale to quantify the patient’s perceived effort.

**Fig 2:**
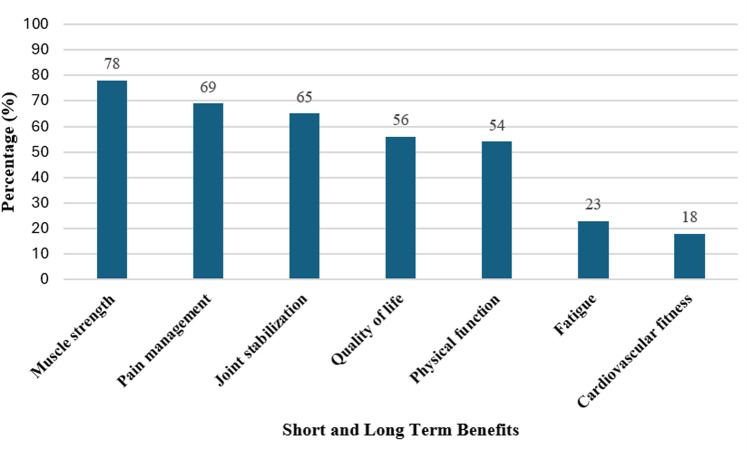
Percentage (%) of websites addressing short- and long-term benefits of exercise training.

**Fig 3:**
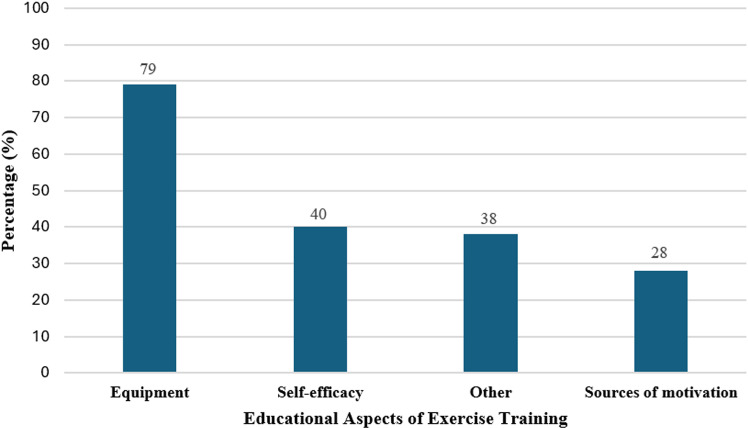
Percentage (%) of websites addressing educational aspects of exercise training. Other: Hydration, sun protection, weather, air quality health index, nutrition/diet, sleep, overcoming kinesiophobia, (i.e., through cognitive behavioural therapy).

Fig 2 illustrates the distribution of websites addressing short- and long-term benefits of exercise training (i.e., muscle strength, pain management, quality of life, physical function, fatigue, and cardiovascular fitness). Fig 3 highlights educational aspects of exercise training (i.e., exercise self-efficacy, equipment, motivational resources, and nutrition needs) that were included in the online resources. Muscle strength (78%) was highlighted as the most common short/long-term benefit of exercise training, whereas cardio-metabolic fitness (18%) was not commonly addressed. A few websites described how support from family and friends increases adherence with exercise programs, while fitness tracking applications were described as important for fostering engagement.

### Website quality

#### DISCERN and GQS.

The median modified DISCERN score (out of 5) was 4 [3–4]. Question 1 on clarity of aims and achievements received the highest rating, while question 5 on areas of uncertainty received the lowest scores (S3 Table in S1 File). The median GQS (out of 5) was 3 [2.3–4] (S5 Table in S1 File). The mean JAMA benchmark criteria score was 3 [2–3] (S4 Table in S1 File). Most websites contained ownership disclosures (94%) and had updated information (90%). Half of the websites provided references to support the content (50%), but only 32% contained authorship or proper citations. Moderate-strong Spearman correlations were observed between total content scores and GQS (rho = 0.76), as well as total content scores and DISCERN (rho = 0.52), p < 0.001 for both.

#### PEMAT.

The mean PEMAT understandability score was 85% ± 12% (≥ 70% reference standard) (S6 Table in S1 File). When applicable, 100% of websites used an active voice. Only 42% of websites provided visual aids with clear titles or captions. The mean PEMAT actionability score was 69% ± 23% (≥ 70% reference standard). Most websites (99%) clearly identified at least one action the user could undertake, but fewer websites (56%) broke down actions into manageable, explicit steps.

### Website readability

The average FKGL and FEAS were 11.0 ± 2.7 and 43.6 ± 10.0, respectively. All of the websites (n = 78, 100%) exceeded a grade 6 level [49,50], which is the recommended grade level for patient educational materials.

## Discussion

This is the first study to characterize internet resources on exercise and physical activity for individuals with EDS/G-HSD, and to assess their content, quality, and readability. These findings are important as we move beyond the COVID-19 pandemic, with a growing emphasis on virtual and hybrid EDS-specific self-management programs that integrate exercise science, rehabilitation, and health psychology to enhance quality of life. This study identified that content differed across websites and quality was moderate-to-good with above recommended reading levels. Increasing the accessibility of high-quality online resources for individuals with EDS/G-HSD, family members, and allied healthcare professionals may provide numerous short and long-term benefits.

Websites are meant to facilitate information transfer. However, most websites were educational (82%), highlighting an opportunity to develop more instructional websites or embedded videos. The mean PEMAT actionability score was 69% ± 23%, which is just below the 70% threshold commonly accepted with this instrument [44]. Most websites identified at least one actionable step, with over half breaking down these actions into explicit guidance. This structure can boost confidence as individuals with EDS/G-HSD often have kinesiophobia or have had negative exercise experiences. Further, while over half of websites incorporated visual aids, only about one-third had clear titles or captions. These elements are important as detailed written instructions, captioned diagrams or videos, and summary tables can make content more manageable, and help promote lifestyle and behavioural changes [51–53].

Muscle strength followed by joint stabilization were the most discussed short- and long-term benefits of exercise training, whereas cardio-metabolic fitness was rarely addressed. However, improving cardiovascular fitness can help control dysautonomia [54], maintain a healthy weight to minimize stress on joints [55] and improve muscle strength, all of which may help reduce chronic pain and fatigue [56]. Pain is an important contributor to quality of life, and is associated with impairments in daily function [57] and mental health conditions, such as anxiety, depression, and panic disorder [58,59]. Other limitations to quality of life that were discussed on some websites include frequent joint dislocations and injuries, frustration arising from challenges in receiving proper diagnosis and management, and symptoms interfering with employment and social activities [59].

The recommendation for patient education materials is that it does not exceed a grade 6 level [53,57]. However, the mean website readability was a grade 11 level which is similar to other website-based research studies [31,60,61]. A high website reading level can limit understanding, reduce treatment adherence, and increase risk of adverse events from exercise training [53]. Thus, this issue is important as individuals with EDS/G-HSD may turn to online resources for information, partly due to reduced awareness of EDS management by health care providers [62].

Our evaluation of online resources highlighted approaches that may be beneficial in promoting exercise training. Specifically, websites that provide examples of exercises that draw similarities with daily living activities, discuss injury prevention strategies (i.e., warm-up and cool-down), address psychological supports, and/or provide a rationale behind certain exercise modalities were deemed to be beneficial for exercise. Further, to promote understandability and actionability of online resources, the inclusion of visual aids, collaboration with international societies and medical professionals for better alignment with patient priorities, and fostering engagement with online resources could be incorporated. Future developments could leverage artificial intelligence to filter or rank search engine results to create a database of websites with the highest content and quality ratings, while user-generated ratings could be displayed to account for individual experiences. Further, a ‘plain language’ extension could be utilized to provide definitions for medical terms and exercise training diagrams to enhance comprehension [52,53].

There were several study limitations. First, we only evaluated websites in English and focused on the first 175 websites for each search term. We selected this approach given that most individuals do not access lower ranked websites beyond the first few pages [63]. Second, a U.S Internet Protocol address was used for the search; thus, the websites analyzed may be different from commonly used websites globally. Third, although exercise training is commonly described in the literature for individuals with EDS/G-HSD, there is no standardized, clinical practice guidelines [4]. It is possible that we may have not captured all the necessary elements in our content score, although our criteria was developed from consensus guidelines [13,15–17,32–40] and expertise from the collaboration of an international group of EDS/G-HSD healthcare professionals.

## Conclusion

In conclusion, our analysis provides insight on the current state of online exercise-training resources for individuals with EDS/G-HSD. While website content varied, specific elements of exercise training principles, safety considerations, and education for self-efficacy were not consistently addressed. Website quality was moderate-to-good but many lacked references and authorship details reducing their credibility. Readability levels exceeded recommended thresholds, potentially limiting accessibility for individuals with lower health literacy. To better support EDS/G-HSD use of online resources given the increased uptake with virtual care, online content should prioritize evidence-based instruction rather than purely educational content, incorporation of videos and specific actionable guidance. Increasing the accessibility of high-quality, user-friendly online resources may improve exercise adoption, in turn enhancing pain management, energy levels, and overall quality of life.

## Supporting information

S1 File S1–S6 Tables provide additional data and analyses for the methods and results. (DOCX)
