# Supplementary material for: Characterizing the content and quality of internet resources on exercise training in Ehlers-Danlos Syndromes and generalized hypermobility spectrum disorder
Source: PLoS One. 2025 Jun 26;20(6):e0325709. doi: 10.1371/journal.pone.0325709 (PMC12200703; doi:10.1371/journal.pone.0325709)
Supplement: S1 File — (DOCX) [file pone.0325709.s001.docx]

**Characterizing the Content and Quality of Internet Resources on Exercise Training in Ehlers-Danlos Syndromes and Generalized Hypermobility Spectrum Disorder**

Jillian Dhawan^1,2^, Sahar Sohrabipour^1,2^, Ali Salman Al-Timimi^1,2^, Brenawen Elangeswaran^1,2^, Omer Choudhary^1,2^, Noor Al Kaabi^1,2^, Megha Ibrahim Masthan^1,2^, Daniel Santa Mina^,3,4,5^, Laura McGillis^3^, Wing Ting Truong ^3^, Encarna Camacho Perez^1,2^, Jane Schubart^6,7^, Mark Lavallee^8^, Timothy Sheehan^9^, Neyha Cherin^10^, Nimish Mittal^3,4,5^, Hance Clarke^,3,5^, Rebecca Bascom^7,9^, Dmitry Rozenberg^1,2,3^

^1^Toronto General Hospital Research Institute, University Health Network, Toronto, ON, Canada.

^2^ Division of Respirology, Temerty Faculty of Medicine, University Health Network, University of Toronto, Toronto, ON, Canada

^3^GoodHope Ehlers-Danlos Syndrome Clinic, University Health Network, Toronto, ON, Canada.

^4^Faculty of Kinesiology and Physical Education, University of Toronto, Toronto, ON, Canada.

^5^Department of Anesthesia and Pain Management, University Health Network, Toronto, ON, Canada.

^6^ Department of Surgery, Penn State College of Medicine, Hershey, PA, USA

^7^Department of Public Health Sciences, Penn State College of Medicine, Hershey, PA, USA

^8^ Department of Orthopedics, UPMC Central Pennsylvania, Harrisburg, PA, USA

^9^ Department of Medicine, Penn State College of Medicine, Hershey, PA, USA

^10^ Department of Physical Medicine and Rehabilitation, Penn State College of Medicine, Hershey, PA, USA

**_________ Supplemental Material _________**

**Supplemental Material**

**Supplementary Table 1:** Website uniform resource identifier from Initial Search on July 14, 2023. (p. 3-5)

**Supplementary Table 2:** Website uniform resource identifier from Updated Search including websites from July 14, 2023, to July 2, 2024. (p. 6)

**Supplementary Table 3:** Modified DISCERN score. (p. 7)

**Supplementary Table 4:** JAMA Benchmark score. (p. 8)

**Supplementary Table 5.** Global Quality Scale score. (p.9)

**Supplementary Table 6:** Patient Education Material and Assessment Tool for Printable Materials for websites. (p. 10-11)

**Supplementary Table 1:** Website uniform resource identifier from Initial Search on July 14, 2023.

| **Website uniform resource identified (search term 1: Ehlers-Danlos Syndrome and Exercises)** | **Rank Google®** |
| --- | --- |
| <https://ehlersdanlosnews.com/eds-and-exercise/#:~:text=Suitable%20exercises,well%20for%20some%20EDS%20patients> | 1 |
| <https://www.ehlers-danlos.org/information/exercise-and-movement-for-adults-with-hypermobile-ehlers-danlos-syndrome-and-hypermobility-spectrum-disorders/> | 2 |
| <https://patientworthy.com/2017/11/21/dos-donts-exercising-with-ehlers-danlos-syndrome/> | 4 |
| <https://connect.mayoclinic.org/blog/ehlers-danlos-syndrome/newsfeed-post/exercise-for-heds-and-hsd/> | 7 |
| <https://www.fitnessblender.com/articles/ehlers-danlos-syndrome-symptoms-treatment-and-exercise> | 8 |
| <https://www.tgfitness.com/is-exercise-good-for-ehlers-danlos-syndrome/> | 9 |
| <https://www.strength-space.com/best-exercises-for-ehlers-danlos/> | 10 |
| <https://www.ehlers-danlos.com/wp-content/uploads/2022/12/Muldowney-The-Muldowney-Protocol-2019s.pdf> | 12 |
| <https://www.strength-space.com/building-stability-strength-with-ehlers-danlos-syndrome/> | 13 |
| <https://massagefitnessmag.com/massage/what-exercises-are-good-for-hypermobility-and-ehlers-danlos-syndrome-eds/> | 14 |
| <https://parrpt.com/your-path-to-strength-and-stability-personalized-exercises-for-eds/> | 15 |
| <https://chelseyengel.medium.com/breaking-down-the-barriers-to-exercising-with-eds-and-hypermobility-692081adc462> | 17 |
| <https://www.physio-pedia.com/Ehlers-Danlos_Syndrome> | 19 |
| <https://bootcampmilitaryfitnessinstitute.com/injury/exercise-ehlers-danlos-syndromes-eds-part-one/exercise-ehlers-danlos-syndromes-eds-part-five/> | 21 |
| <https://www.surreyphysio.co.uk/top-5/best-5-exercises-for-ehlers-danlos-syndrome/> | 22 |
| <https://www.movewelldaily.com/top-5-heds-and-hypermobility-considerations-movement-exercise/> | 26 |
| <https://back-in-business-physiotherapy.com/what-we-treat/ehlers-danlos-syndrome.html> | 29 |
| <https://www.negenetics.org/gemss/conditions/ehlers-danlos-syndrome-eds/physical-activity-trips-events> | 30 |
| <https://alanspanosmd.com/wp-content/uploads/2022/03/Physical-Therapy-Exercise-Braces-for-People-with-EDS.pdf> | 35 |
| <https://www.caringmedical.com/prolotherapy-news/5-tips-working-hypermobility-syndrome/> | 37 |
| <https://www.easyexercising.com.au/how-easy-exercising-can-help-with-ehlers-danlos-syndrome-eds/> | 38 |
| <https://www.wellnessthroughmovement.com.au/blog/ehlers-danlos-syndrome-eds-awareness-month> | 42 |
| <https://www.howwedidthat.com/ehlers-danlos#/> | 44 |
| <https://www.hypermobility.org/exercise> | 45 |
| <https://www.fightveds.org/diet-exercise> | 46 |
| <https://www.calibratepilates.com/blog/clinical-conversation-ehlers-danlos> | 49 |
| <https://www.btetechnologies.com/therapyspark/ehlers-danlos/> | 50 |
| <https://www.hydroworx.com/blog/ehlers-danlos-syndrome-patient-gaining-strength-aquatic-therapy/> | 51 |
| <https://www.exercisethought.com/blog/hypermobility> | 53 |
| <https://www.thefibroguy.com/blog/hypermobility-knee-exercises/> | 54 |
| <https://en.wikibooks.org/wiki/Exercise_as_it_relates_to_Disease/The_role_of_strength_training_in_managing_Ehlers_Danlos> | 56 |
| <https://webspace.clarkson.edu/~lrussek/docs/hypermobility/Russek_HSD104.pdf> | 57 |
| <https://integrativewellnesspt.com/2017/12/05/eds-tips-water-exercise-suggestions-for-success/> | 63 |
| <https://www.thefemword.world/the-word/ehlers-danlos-syndrome-and-its-impact-on-life> | 70 |
| <https://stretchaffect.com/blog/hypermobility-exercises-structure/> | 71 |
| <https://integrativemvmt.com/hypermobilitystrength/> | 77 |
| <https://www.theilcfoundation.org/managing-eds/> | 82 |
| <https://now.aapmr.org/ehler-danlos-syndrome/> | 85 |
| <https://www.advanced-rehabilitation.com/blog/l5vlgmxaytz86k466t7hyf2k30akap> | 87 |
| <https://www.therapeuticassociates.com/certified-hand-therapy-for-ehlers-danlos-syndrome-or-hypermobility/> | 90 |
| <https://restoremotion.com/ehlers-danlos-syndrome-eds/> | 91 |
| <https://www.nhs.uk/conditions/joint-hypermobility-syndrome/> | 93 |
| <https://www.chronicpainpartners.com/dr-mark-lavallee-webinar-exercise-is-medicine-for-ehlers-danlos-on-march-3-2015/> | 94 |
| <https://physiostore.ca/ehlers-danlos-syndrome-exercise-blog/> | 95 |
| <https://www.drjaimebrainerd.com/health/acceptable-exercises-for-people-with-ehlers-danlos-syndrome-eds/> | 96 |
| <https://edswellness.org/edsfittip-physical-therapy-exercise-help-strengthen-shoulders-core-neck-gluts/> | 98 |
| <https://www.aafp.org/pubs/afp/issues/2021/0415/p481-s1.html> | 99 |
| <https://draxe.com/health/ehlers-danlos-syndrome/> | 100 |
| <https://gmb.io/hypermobility/> | 103 |
| <https://utswmed.org/medblog/ehlers-danlos-diagnosis-care/> | 114 |
| <https://www.stylist.co.uk/fitness-health/workouts/hypermobility-fitness-work-out-injury/579174> | 120 |
| <https://cdn.ymaws.com/www.ohiopt.org/resource/resmgr/files/Annual_Conference_2017/Handouts/ehlers_danlos_syndrome.pdf> | 123 |
| <https://chronicallyawesome.org.uk/exercise-and-hypermobility/> | 125 |
| <https://www.allwayswell.com/blog/exercise-and-ehlers-danlos-syndrome-by-brian-kitzerow-dpt-cmpt-ocs> | 128 |
| **Website uniform resource identified (search term 2: Ehlers-Danlos Syndrome and Physical Activity)** | **Rank Google®** |
| <https://www.ehlers-danlos.org/information/physical-therapy-for-hypermobility/> | 4 |
| <https://patient.info/bones-joints-muscles/ehlers-danlos-syndrome-leaflet> | 36 |
| <https://www.thesports.physio/hypermobility-and-sport/> | 42 |
| <https://www.practicalpainmanagement.com/pain/other/hypermobile-ehlers-danlos-syndrome-update-therapeutic-approaches-pain-management> | 51 |
| <https://www.facebook.com/ehlers.danlos/videos/pain-free-fitness-with-mariah-heller/449846350306616/> | 53 |
| <https://www.versusarthritis.org/about-arthritis/conditions/joint-hypermobility/> | 73 |
| <https://www.bodyfitphysio.com.au/physiotherapy-conditions/hypermobility/ehlers-danlos-syndromes-eds-hypermobility-spectrum-disorder-hsd/> | 74 |
| <https://orthop.washington.edu/patient-care/articles/arthritis/ehlers-danlos-syndrome.html> | 113 |
| <https://www.muldowneypt.com/ehlers-danlos-syndrome-information/> | 128 |
| <https://progressiveptandrehab.com/joint-hypermobility-ehlers-danlos-syndrome/> | 129 |
| <https://charmaustin.com/ehlers-danlos-syndrome-eds/> | 131 |

**Supplementary Table 2:** Website uniform resource identifier from Updated Search including websites from July 14, 2023, to July 2, 2024.

| **Website uniform resource identified (search term 1: Ehlers-Danlos Syndrome and Exercises)** | **Rank Google®** |
| --- | --- |
| <https://www.hingehealth.com/resources/articles/ehlers-danlos-syndrome/> | 1 |
| <https://jeanniedibon.com/the-truth-about-exercising-with-hypermobility-and-ehlers-danlos/> | 4 |
| <https://www.thefibroguy.com/blog/hypermobility-and-exercise-part-1/> | 5 |
| <https://petersenpt.com/physical-therapy-for-ehlers-danlos-syndrome> | 8 |
| <https://www.ehlers-danlos.com/physical-therapy/#1706025876219-1b9674a7-3add> | 9 |
| <https://lotusptny.com/blog/ehlers-danlos-syndrome-physical-therapy-managing-joint-instability> | 10 |
| <https://www.longdom.org/open-access/the-role-of-physical-therapy-in-ehlersdanlos-syndrome-management-104095.html> | 17 |
| <https://empoweralaska.com/physical-therapy-treatments/ehlers-danlos-syndrome/> | 18 |
| <https://www.pittsburghphysmed.com/blog/navigating-the-challenges-of-ehlers-danlos-syndrome-a-comprehensive-guide-to-physical-therapy> | 20 |
| <https://notjustbendy.com/tag/ehlers-danlos-syndrome/> | 21 |
| <https://www.webmd.com/a-to-z-guides/ehlers-danlos-syndrome-facts> | 23 |
| **Website uniform resource identified (search term 2: Ehlers-Danlos Syndrome and Physical Activity)** | **Rank Google®** |
| <https://www.actifypt.com/post/6-ways-physical-therapy-can-help-with-eds-hsd-pain> | 18 |
| <https://www.choosept.com/guide/physical-therapy-guide-benign-hypermobility-joint-syndrome> | 21 |

**Supplementary Table 3**: Modified DISCERN score.

| Description | Score | Websites (n=78) |
| --- | --- | --- |
| 1. Are the aims clear and achieved? | Yes= 1 point, No= 0 point | 68 (87%) |
| 1. Are reliable sources of information used? | Yes= 1 point, No= 0 point | 49 (63%) |
| 1. Is the information presented balanced and unbiased? | Yes= 1 point, No= 0 point | 66 (85%) |
| 1. Are additional sources of information listed for patient reference? | Yes= 1 point, No= 0 point | 62 (79%) |
| 1. Are areas of uncertainty mentioned? | Yes= 1 point, No= 0 point | 34 (44%) |

**Supplementary Table 4:** JAMA Benchmark score.

| Score | Description | Websites |
| --- | --- | --- |
| **1** | Authorship: Proper citations used in the website | 25/78 (32%) |
| **2** | Attribution: References and sources of information are identified | 39/78 (50%) |
| **3** | Currency: Website updated with latest information | 47/52 (90%) |
| **4** | Disclosure: Website ownership, advertising, sponsorship and conflicts of interest are disclosed | 73/78 (94%) |

Note: A denominator less than 78 is used when the criteria were not applicable to a website(s).

**Supplementary Table 5:** Global Quality Scale Score.

| Global Score | Description | Websites (n=78) |
| --- | --- | --- |
| 1 | Poor quality, poor flow, most information missing, not useful for patients | 1 (1%) |
| 2 | Generally poor quality, poor flow, some information given but many important topics missing, of very limited use to patients | 19 (24%) |
| 3 | Moderate quality, suboptimal flow, some important information is adequately discussed but others poorly discussed, somewhat useful for patients | 26 (33%) |
| 4 | Good quality, good flow, most relevant information is covered but some topics not covered, useful for patients | 24 (31%) |
| 5 | Excellent quality, excellent flow, very useful for patients | 8 (10%) |

Note: The percentage values have been rounded, thus, may not add up to 100%.

**Supplementary Table 6:** Patient Education Material and Assessment Tool for Printable Materials for websites

**Understandability**

| Item # Item Websites | | |
| --- | --- | --- |
| Topic: Content | | |
| 1 | The material makes the purpose completely relevant. | 69/78 (88%) |
| 2 | The material does not include information or content that distracts from its purpose. | 72/78 (92%) |
| Topic: Word Choice & Style | | |
| 3 | The material uses common, everyday language. | 73/78 (94%) |
| 4 | Medical terms are used only to familiarize audience with the terms. When used, medical terms are defined. | 72/78 (92%) |
| 5 | The material uses the active voice. | 78/78 (100%) |
| Topic: Use of Numbers | | |
| 6 | Numbers appearing in the material are clear and easy to understand. | 37/41 (90%) |
| 7 | The material does not expect the user to perform calculations. | 73/77 (95%) |
| Topic: Organization | | |
| 8 | The material breaks or “chunks” information into short sections. | 75/76 (99%) |
| 9 | The material’s sections have informative headers. | 72/75 (96%) |
| 10 | The material presents information in a logical sequence. | 72/75 (96%) |
| 11 | The material provides a summary. | 27/51 (53%) |
| Topic: Layout & Design | | |
| 12 | The material uses visual cues (e.g. arrows, boxes, bullets, bold, larger font, highlighting) to draw attention to key point. | 67/75 (89%) |
| Topic: Use of Visual Aids | | |
| 13 | The material uses visual aids whenever they could make content more easily understood (e.g. illustration of healthy portion size). | 42/77 (55%) |
| 14 | The material’s visual aids reinforce rather than distract from the content. | 40/61 (66%) |
| 15 | The material’s visual aids have clear titles or captions. | 25/60 (42%) |
| 16 | The material uses illustrations and photographs that are clear and uncluttered. | 50/62 (81%) |
| 17 | The material uses simple tables with short and clear row and column headings. | 9/21 (43%) |

*Total Points***:** _____________ *Total Possible Points***:** _____________
**Understandability Score (%):** _____________

(Total Points / Total Possible Points x 100)

Note: A denominator less than 78 is used when the criteria was not applicable to a website(s).

**Actionability**

| Item # | Item | Website |
| --- | --- | --- |
| 20 | The material clearly identifies at least one action the user can take. | 77/78 (99%) |
| 21 | The material addresses the user directly when describing actions. | 65/78 (83%) |
| 22 | The material breaks down any action into manageable, explicit steps. | 44/78 (56%) |
| 23 | The material provides a tangible tool (e.g. menu planners, checklists) whenever it could help the user take action. | 29/78 (37%) |
| 24 | The material provides simple instructions or examples of how to perform calculations. | 12/12 (100%) |
| 25 | The material explains how to use the charts, graphs, tables or diagrams to take actions. | 20/25 (80%) |

*Total Points***:** _____________
*Total Possible Points***:** _____________
**Actionability Score (%):** _____________
(Total Points / Total Possible Points x 100)

Note: A denominator less than 78 is used when the criteria was not applicable to a website(s).
